# Hyponatremia: Special Considerations in Older Patients

**DOI:** 10.3390/jcm3030944

**Published:** 2014-08-18

**Authors:** Roy L. Soiza, Kirsten Cumming, Jennifer M. Clarke, Karen M. Wood, Phyo K. Myint

**Affiliations:** 1 Department of Medicine for the Elderly, NHS Grampian, c/o Wards 303/4, Aberdeen Royal Infirmary, Foresterhill, Aberdeen AB25 2ZN, UK; E-Mail: phyo.myint@abdn.ac.uk; 2 School of Medicine & Dentistry, University of Aberdeen, Aberdeen AB25 2ZD, UK; E-Mails: kirsten.cumming.09@aberdeen.ac.uk (K.C.); jennifer.clarke.10@aberdeen.ac.uk (J.M.C.); karen.wood.10@aberdeen.ac.uk (K.M.W.)

**Keywords:** aging, arginine vasopressin, geriatrics, hyponatremia, old, salt, sodium

## Abstract

Hyponatremia is especially common in older people. Recent evidence highlights that even mild, chronic hyponatremia can lead to cognitive impairment, falls and fractures, the latter being in part due to bone demineralization and reduced bone quality. Hyponatremia is therefore of special significance in frail older people. Management of hyponatremia in elderly individuals is particularly challenging. The underlying cause is often multi-factorial, a clear history may be difficult to obtain and clinical examination is unreliable. Established treatment modalities are often ineffective and carry considerable risks, especially if the diagnosis of underlying causes is incorrect. Nevertheless, there is some evidence that correction of hyponatremia can improve cognitive performance and postural balance, potentially minimizing the risk of falls and fractures. Oral vasopressin receptor antagonists (vaptans) are a promising innovation, but evidence of their safety and effect on important clinical outcomes in frail elderly individuals is limited.

## 1. Introduction

Hyponatremia is the commonest electrolyte imbalance encountered in clinical practice [1]. It is associated with multiple poor clinical outcomes including increased mortality [2], increased length of hospital stay [3,4] and institutionalization [4]. Hyponatremia occurs due to disruption of sodium and water homeostasis, normally maintained by complex multi-system physiological mechanisms [5]. It represents an excess of water relative to sodium, though both sodium and total body water may be increased, normal or diminished. Consequently, there are numerous potential underlying causes of hyponatremia, spanning a broad spectrum of diseases, pharmacotherapy and pathophysiological variants each with different treatment requirements. This review examines some of the recent insights into the special implications of hyponatremia in the older patient, paying particular attention to the potential for even mild, chronic hyponatremia to result in subtle symptoms such as cognitive impairment, bone demineralization, falls and fractures [6,7,8]. The unique challenges of accurate diagnosis and treatment of the underlying causes in the older patient are also highlighted.

## 2. Epidemiology

Prevalence of hyponatremia is known to increase in frail patient groups, particularly elderly patients where hyponatremia is observed in almost half of acute geriatric admissions [9,10]. Older people have an increased predisposition to hyponatremia due to degenerate physiology, multiple co-morbidities and polypharmacy [5]. Hospitalized older people have a further susceptibility to hyponatremia due to dehydration, inappropriate fluid therapy and iatrogenic interventions.

Hyponatremia in elderly patients is predominantly mild and chronic (*i.e.*, serum sodium 130–134 mmol/L developing over >48 h) [11]. These cases are classically devoid of the obvious neurological symptoms seen in acute severe hyponatremia due to homeostatic compensatory mechanisms which allow brain cells to adapt to changes in plasma osmolality within hours. As a result, mild, chronic hyponatremia has typically been considered to be asymptomatic despite being associated with major geriatric presentations (e.g., falls and confusion), and multi-organ pathological changes. Also, in the absence of obvious neurological symptoms, mild, chronic hyponatremia is at risk of being overlooked. The older person’s increased risk of both hyponatremia and its subsequent clinical sequelae makes hyponatremia in elderly patients of particular clinical importance.

## 3. Does Hyponatremia Cause Cognitive Impairment?

One of the symptoms often associated with hyponatremia is cognitive impairment, particularly in elderly populations. In cases of acute hyponatremia, the pathological mechanisms involved are well understood, with the neurological problems thought to be due to cerebral edema and hyponatremic encephalopathy [12]. However, understanding of such mechanisms in the more prevalent, chronic hyponatremia is poor. Several studies have recently found an association between cognitive impairment and chronic hyponatremia, even at a mild to moderate level.

In a study examining performance in a battery of cognitive function tests, Renneboog *et al.* [6] found that hyponatremic participants had a median response time of 673 ms across the various assessments. Each participant acted as their own control, and when participants were normonatremic, the median response time was found to be significantly reduced at 615 ms (*p* < 0.001). Similarly, Vaghasiya *et al.* [13] conducted a study evaluating the performance of hyponatremic participants in the Mini Mental State Examination (MMSE), with the assessment being repeated again after correction of the electrolyte disorder in the same participants. There was an increase in MMSE score following improvement in serum sodium concentration in 93% of patients (*p* = 0.001). As the only aspect of the investigations that varied was the serum sodium level, the findings from both of these studies strongly suggest that the correction of hyponatremia is the factor causing the improved cognitive performance. Although a learning effect cannot be excluded, the MMSE is robust in repeat testing and shows little susceptibility to learning effects [14,15]. Moreover, in the study by Renneboog *et al.* [6], the possibility of a learning effect was eliminated as half of the participants tested initially had normal serum sodium levels while the remaining participants were tested first during a period of hyponatremia. Therefore, it seems likely that hyponatremia itself leads to cognitive impairment, and, additionally, that correction of hyponatremia can lead to improved cognition. However, some caution is warranted as the study by Renneboog *et al.* was unblinded and Vaghasiva *et al.* have not fully published their results.

Gunathilake *et al.* [16] used the Audio Recorded Cognitive Screening Tool to compare the cognition of hyponatremic participants to a control group. They concluded that the scores of the group with mild hyponatremia were on average 4.67 units lower than the control group (*p* = 0.01, 95% CI 1.56–7.79). Interestingly, the significant reduction in cognitive performance occurred when sodium levels decreased by as little as 5 mmol/L.

Despite these studies using different methods of assessing cognition, all consistently found that cognitive impairment occurs in patients with chronic hyponatremia. However, the mechanisms explaining this association remain unclear. In chronic hyponatremia, serum sodium levels decline gradually, allowing the body time to adapt. To prevent swelling initially, the glial cells use the Na^+^-K^+^-ATPase system to move sodium out of cells whilst also expelling osmolytes [17]. This adaptation results in water leaving the brain following an osmotic gradient, preventing the accumulation of fluid in cells and thus preserving function [18]. Therefore, any cognitive impairment associated with chronic hyponatremia is due to a different pathological mechanism than is the case for acute hyponatremia.

Potentially, much of the cognitive impairment may be secondary to the conditions causing hyponatremia. For example, liver cirrhosis is known to be one of the causes of chronic hyponatremia and 62% of patients with this condition score lower than expected on psychometric testing, even when normonatremic [19]. Further research is needed into the mechanisms by which chronic hyponatremia leads to cognitive impairment.

## 4. Hyponatremia and Bone Demineralization

### 4.1. Non-Randomised Rodent Studies

Hyponatremia is also reputed to lead to bone demineralization and fractures. Verbalis *et al.* [8] used a rat model of syndrome of inappropriate anti-diuretic hormone (SIADH) to measure bone demineralization in six week old male rats compared to a control group of normonatremic rats. Mean serum sodium concentration [sNa^+^] of the hyponatremic and normonatremic groups were 110 ± 2 mmol/L and 141 ± 1 mmol/L respectively. Following three months in a hyponatremic state, the rats had a 30% reduction in femoral bone mineral density (BMD) in comparison to the normonatremic rats (*p* < 0.001). Barsony *et al.* [20] used a similar rodent model as Verbalis *et al.* [8] but, to replicate the effect in the elderly, they used male rats aged 22 months. Fifteen rats were subjected to hyponatremia for 18 weeks with a mean [sNa^+^] of 112.7 ± 1.3 mmol/L and 10 rats were used as controls with a mean [sNa^+^] of 142.7 ± 1.1 mmol/L. A 16% reduction in BMD was seen in the hyponatremic group (*p* < 0.05). Thus, rodent studies by both Verbalis *et al.* [8] and Barsony *et al.* [20] show significant decreases in excised femoral BMD resulting from chronic hyponatremia. The two-fold discrepancy in effect between these two studies is probably due to the aged control rats in the Barsony *et al.* [20] study also losing BMD through the ageing process. 

### 4.2. Case-Control Studies

**Figure 1:**
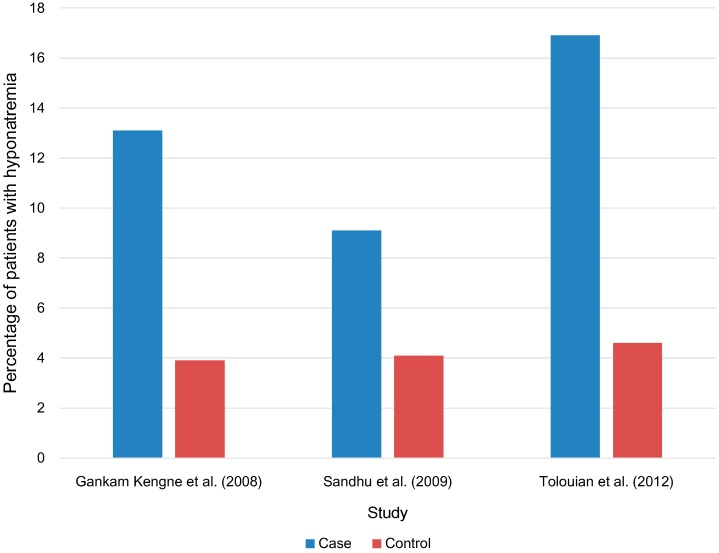
Prevalence of hyponatremia in patients with a bone fracture (case) and without a bone fracture (control).

Several studies have looked at the relationship between hyponatremia and bone fractures. Three case-control studies of individuals aged 65 years or over compared the prevalence of hyponatremia where the case group had a verified bone fracture and the control group had no history of bone fracture (Figure 1). The study by Gankam Kengne *et al.* [7] determined a prevalence of hyponatremia in the case and control groups of 13.1% and 3.9% respectively (*p* < 0.01) (Figure 1). Two other studies found the prevalence of hyponatremia to be 9.1% and 16.9% in the case groups compared with 4.1% and 4.6% in control groups (*p* = 0.007 and *p* = 0.03) [21,22] (Figure 1).

For the association between hyponatremia and bone fractures, adjusted ORs of 4.16 and 4.80 were determined (95% CI: 2.24–7.71 and 1.06–21.67) [7,22]. The first OR was adjusted for possible confounding variables identified through univariate analysis as having *p* < 0.05 and the other was adjusted for age only.

The results from these case-control studies demonstrate a higher prevalence of hyponatremia in the case group than in the control group as well as a strong relationship between hyponatremia and bone fractures after adjusting for potential confounding variables. However, deductions about the relationship between hyponatremia and osteoporosis cannot be made from these case-control studies because they do not provide BMD data.

### 4.3. Cross-Sectional Cohort Studies

Three cross-sectional cohort studies assessed the relationship between hyponatremia and a reduction in BMD in humans and all had a mean age greater than 60 years. The study by Verbalis *et al.* [8] used information from the Third National Health and Nutrition Examination Survey. 5598 subjects were normonatremic whereas the exact number of individuals who were hyponatremic was not provided. Kinsella *et al.* [23] undertook a cohort study involving 1408 females. There were 254 verified fractures and 4.2% (59 individuals) were hyponatremic. Of the subjects with a fracture, 8.7% were hyponatremic whereas only 3.2% without a fracture were hyponatremic. The study by Hoorn *et al.* [24] involved 5208 patients, of which 7.7% (399 individuals) had hyponatremia.

Verbalis *et al.* [8] demonstrated a significant positive linear relationship between femoral BMD and [sNa^+^] in the hyponatremic subjects (*p* < 0.01) whereas no relationship was seen in the normonatremic individuals (*p* = 0.99). This correlation was further established as the ORs for hyponatremia and osteoporosis at the femoral neck and total hip (defined by BMD with *T*-score < −2.5) were 2.87 and 2.85 (95% CI: 1.41–5.81 and 1.03–7.86). These results substantiate an association between hyponatremia and osteoporosis. An association between hyponatremia and osteoporosis was also demonstrated in the study by Kinsella *et al.* [23]. They reported a significant decrease in BMD in the hyponatremic subjects when compared to the normonatremic subjects, with mean *T*-scores of −2.6 and −2.3 (*p* = 0.03) respectively. Hyponatremic individuals also had a significantly higher prevalence of osteoporosis of 57.6% compared with 44.3% in controls (*p* = 0.04). Hoorn *et al.* [24] did not find a correlation between hyponatremia and BMD in either the lower femoral neck or the lumbar spine after adjustment for age and sex (*p* = 0.105 and *p* = 0.473). Kinsella *et al.* [23] established a fully adjusted OR for the relationship between hyponatremia and bone fractures of 2.25 (95% CI: 1.24–4.09) (details of adjusted factors are shown in Table 1). As this was adjusted for *T*-score, this shows that the increased risk of bone fractures caused by hyponatremia occurs independently of BMD.

Hoorn *et al.* [24] established a fully adjusted OR of 1.61 for the association between hyponatremia and vertebral fractures (95% CI: 1.00–2.59). For non-vertebral fractures, an adjusted OR of 1.34 was found (95% CI: 1.08–1.68) (adjustments shown in Table 1). Consequently, these results show a positive correlation between hyponatremia and both non-vertebral and vertebral fractures.

**Table 1:** Factors adjusted for in models in the cohort studies.

Study	Adjustments
Verbalis *et al.* (2010) [8]	age (years), sex, body mass index, history of diuretic use, physical activity, serum 25-hydroxy-vitamin D levels
Kinsella *et al.* (2010) [23]	age (years), *T*-score, chronic kidney disease stage, osteoporotic risk factors (amenorrhoea, low dietary calcium (Ca^2+^) intake, high alcohol intake, maintenance steroids, ever having smoked, family history of osteoporosis and history of liver disease) and osteoporosis treatment (use of Ca^2+^, vitamin D, antiresorptive therapy and hormonal replacement therapy)
Hoorn *et al.* (2011) [24]	age (years), sex, body mass index, disability index, use of diuretics or psycholeptics, recent falls and prevalent diabetes mellitus

### 4.4. Falls

The work of Renneboog *et al.* [6] indicates that chronic hyponatremia, even when mild, is related to an increased risk of falls. They determined an adjusted OR of 67.43 (95% CI: 7.48–607.42) for the relationship between chronic asymptomatic hyponatremia and falling in patients with a mean age of 72 years. They adjusted for age, sex and an array of other potential confounders determined through univariate analysis. Moreover, correction of hyponatremia resulted in improvements in the degree of postural sway.

In summary, human studies have consistently established a relationship between chronic hyponatremia and an increased risk of bone fractures. Chronic hyponatremia leads to decreased BMD in young and old rats but the effects of chronic hyponatremia on BMD in humans is less clear as studies have reported conflicting results. Verbalis *et al.* [8] and Kinsella *et al.* [23] established that hyponatremia was associated with a reduction in BMD, though Kinsella *et al.* [23] reported that hyponatremia caused bone fractures independently of BMD status. Hoorn *et al.* [24] found no relationship between hyponatremia and BMD. Due to the increased risk of falling with hyponatremia, these patients are at a considerably greater risk of sustaining a fracture because of the combined risks of fracture due to hyponatremia (either due to a reduction in BMD or bone quality) and increased risk of falling. 

## 5. Diagnosing the Causes of Hyponatremia

Clinical management of hyponatremia is based on treating the underlying causes (see Table 2) but accurate determination of etiology of hyponatremia is notoriously challenging, particularly in elderly patients [25]. The most widely accepted means of obtaining accurate diagnosis is expert physician’s clinical judgment [26], but relatively few clinicians have a special interest in hyponatremia so this may not be practical. Moreover, a clinical history may be difficult to obtain due to cognitive or sensory impairment and multiple potential causes for hyponatremia may co-exist. Diagnosis of underlying causes depends crucially on accurate assessment of volemic status. However, there is currently no reliable biomarker of hydration for older people [27] and clinical examination is unreliable [27,28].

Accurate appreciation of the etiology of hyponatremia is essential, not only for appropriate clinical management but also to prevent development of hyponatremia. At present, there are few quality studies reporting etiology of hyponatremia in older people. Most reports are from retrospective studies that rely on diagnosis made by non-expert clinicians retrospectively reviewing case notes, which frequently lack sufficient detail to allow accurate diagnosis (*i.e.*, documentation of volemic status, appropriate investigations). The reported commonest cause of hyponatremia is SIADH [29]. However, with the increased prevalence of hyponatremia in older people there is no corresponding increased prevalence of SIADH [30]. This raises concern that SIADH may be over diagnosed, particularly in hypovolemic older people. Diagnosis of SIADH requires strict diagnostic criteria (e.g., urine concentration and sodium measurements). These important investigations are commonly neglected in routine clinical practice and so relevant data are frequently unavailable in observational studies. Moreover, the diagnosis of SIADH depends crucially on patients being in a euvolemic state which cannot be confirmed in the absence of a reliable biomarker of hydration for older people. One study reported that of all the cases of hyponatremia attributed to SIADH, not one fulfilled the SIADH diagnostic criteria [31]. Another group reported that specific etiology of SIADH was identifiable in only 54% of cases of hyponatremia ascribed to SIADH [32]. Whilst many cases of SIADH have no obvious cause, this raises the possibility that some are misdiagnosed. Subsequently patients may be subject to serious clinical consequences as the management of SIADH (fluid restriction) is the exact opposite of the management of hypovolemic hyponatremia (vigorous fluid replacement).

**Table 2:** Main contributory causes of hyponatremia in older people [5].

Hypovolemic hyponatremia
Inadequate fluid intake or replacement
Diuretic therapy
Diarrhea and vomiting
Pancreatitis/third space loss of fluids
Renal salt wasting
Burns and skin losses
Mineralocorticoid deficiency
**Euvolemic hyponatremia**
Syndrome of inappropriate antidiuretic hormone
Medications
Hypothyroidism
Fluid loss with inappropriate fluid (salt) replacement
Glucocorticoid deficiency
**Hypervolemic hyponatremia**
Cardiac failure
Cirrhosis
Chronic kidney disease
Acute kidney injury
Nephrotic syndrome

Supporting the concerns of “inappropriate SIADH diagnoses”, three independent groups have found that the etiology of hyponatremia in older people is predominantly multifactorial, with patients presenting with conflicting signs of hydration [31,32,33]. This further complicates the already challenging task of diagnosing underlying causes of hyponatremia and reinforces the need for a reliable biomarker of hydration in older people [28].

Further investigation of the elderly hyponatremic patient depends on the working diagnosis and readers may find it helpful to refer to recent open-access guidelines, which include a proposed algorithm [11]. However, these are not designed with frail elderly people in mind. For example, although urinalysis is recommended, the guidelines point out results may be misleading in those with chronic kidney disease or diuretics, both of which are highly prevalent in older age groups.

Reportedly common underlying causes of hyponatremia include pharmacotherapies (thiazide and loop diuretics, antidepressants, anticonvulsants, non-steroidal anti-inflammatories, proton pump inhibitors), co-morbidities (congestive cardiac failure, renal failure, cirrhosis, respiratory infections), fluid overload and volume depletion [7,31,32,33,34]. Reports of the pharmacotherapy and polypharmacy associated hyponatremia are increasing. Thiazide diuretic induced hyponatremia has been described as a “silent epidemic” due largely to the combination of their prescription, degenerate renal physiology and concomitant prescription of non-steroidal anti-inflammatories [35]. This is important because of the high prevalence of hypertension in the aging population and widespread thiazide diuretic use. Similarly, the frequent prescribing pattern of other medications known to cause hyponatremia may be important contributing and easily treatable causes of the increased prevalence of hyponatremia in older people. Acknowledging the prevalence of polypharmacy in older patients Movig *et al.* [36] report that concomitant diuretic and selective serotonin reuptake inhibitor (SSRI) therapy incurs a 13.5 increased odds ratio of developing hyponatremia compared to SSRI therapy alone. Conversely, given the high prevalence of multiple co-morbidities and polypharmacy in older people it is likely that causes of hyponatremia attributable to simple hypovolemia are misattributed to other causes [31,33]. Hypovolemia may be the result of over use or incorrect use of diuretics, or inadequate fluid intake or replacement, or a combination of these, which may simply be overlooked in the presence of other potential contributing factors. These etiological reports are promising and suggest that the prevalence of hyponatremia in older people could potentially be reduced by appropriate prescribing patterns, optimization of co-morbidities and adequate fluid therapies. However, in most of these observational studies some important study design limitations should be considered. Frequent omission of appropriate clinical investigations, lack of documentation and/or questionable accuracy of records of volemic state and experience of the diagnosing clinicians may hinder results and cause erroneous diagnosis. These limitations notably reflect how challenging and neglected hyponatremia in elderly patients is in routine clinical practice.

Further research is required to accurately delineate the cause(s) of hyponatremia in older people. Future studies should be prospective and include expert panel review, with completion and full data availability of appropriate investigations. In the meantime establishing a reliable biomarker of hydration for older people is necessary in order to increase accuracy of the diagnostic process. Accurate understanding of etiology of hyponatremia in older people may help prevent and improve clinical management of hyponatremia which could potentially deliver significant health and economic benefits in the form of reduced complications of hyponatremia.

## 6. Treatment of Hyponatremia in Older People

Treatment will depend on the underlying cause, although a recent review article highlighted significant differences in expert panel consensus recommendations for treatment of hyponatremia [37]. Investigation of underlying cause will be the same as younger adults though special attention should be placed upon the possibility of medication-induced hyponatremia and volemic status. Sometimes, the treatment options are obvious, e.g., when hyponatremia is due to hypothyroidism or adrenal insufficiency. The high prevalence of multiple prescribed medications (polypharmacy) means many cases may be related to medications. Hypovolemic hyponatremia is especially common as fluid intake may be impaired by physical and cognitive function and decreased sensitivity of normal physiological thirst and homeostatic mechanisms [5]. Table 3 lists common treatment strategies and a brief synopsis of special considerations in older people.

**Table 3:** Treatment options for hyponatremia.

Treatment	Indication	Special Considerations in Older People
Optimize prescribing	Drug-induced hyponatremia	Medications more likely to be needed due to co-morbidities.
Isotonic saline	Hypovolemic hyponatremia	Hypovolemia common due to immobility, confusion and malnutrition. Assessment of volemic state is notoriously difficult so may worsen hyponatremia if underlying causes are misdiagnosed.
Hypertonic saline	Severe hyponatremia from any cause	Needs expert monitoring. High potential for over-correction.
Fluid restriction	SIADH	Poor tolerability and high failure rate. Will worsen hyponatremia where dehydration is misdiagnosed as SIADH.
Demeclocycline	SIADH	Unpredictability of effect requires special caution due to lower resilience.
Diuretics	Hypervolemic hyponatremia	Can worsen hyponatremia by increasing urinary sodium excretion.
Salt tablets	Hypovolemic hyponatremia	Rarely indicated as dietary sodium intake is usually sufficient and total body sodium is not normally low in most cases of hyponatremia.
Lithium	SIADH	Inconsistent results, with high risk of adverse drug effects and toxicity.
Urea	SIADH	Only available in some countries. Unpalatable. Higher risk of uremia.
Vaptans	SIADH or hypervolemia	Risk of dehydration and over-correction. Expensive. Only licensed for SIADH in Europe.

### 6.1. Optimizing Prescribing

Judicious use of medications and optimal drug prescribing is one of the cornerstones of comprehensive geriatric assessment [38]. Almost three-quarters of elderly patients with hyponatremia after fragility fracture were prescribed at least one medication known to cause hyponatremia [33]. Identification of the culprit medication and de-prescribing or minimizing the dose may be all that is required. As highlighted earlier, common medications implicated in elderly patients include thiazide diuretics and antidepressants. A high prevalence of proton pump inhibitor prescribing has been described in two recent and independent studies of older people with fragility fractures and hyponatremia [33,39]. It is not known if stopping these agents can effectively prevent or treat hyponatremia, but this should be carefully considered because they are arguably over-used. Almost two-thirds of hospitalized older people prescribed proton pump inhibitors do not have a valid indication for their use [40].

### 6.2. Fluid Therapy or Restriction

Since hyponatremia is usually a disorder of fluid balance rather than pure salt depletion, correction of volemic status is a mainstay of treatment. Fluid restriction, typically to about 800 mLs in 24 h, has long been the first line treatment of SIADH. It may be more acceptable in older people than younger patients but is often ineffective or poorly tolerated nevertheless. It can take a long time to be effective and is prone to failure due to hidden liquids in foods and discomfort from thirst. Moreover, in SIADH, there is a downward re-setting of the “thirst osmostat” and a lower plasma osmolality than normal will trigger thirst and increased fluid intake [41].

Intravenous fluid resuscitation is indicated in all forms of hypovolemic hyponatremia. In hypovolemic hyponatremia, isotonic saline suffices and will often lead to very rapid sodium increase, as non-osmotic vasopressin secretion is rapidly suppressed. Most experts recommend using only isotonic saline (0.9% solution of sodium chloride), but boluses of hypertonic solutions are often used in acute and/or severe hyponatremia. No more than 100 mLs of 3% saline are recommended, though these can be repeated as needed [26]. The latter requires intensive monitoring as there is a risk of osmotic demyelination with excessively rapid correction. Corrections below 4 mmol/L in 24 h are associated with poorer outcome [42] but the maximum rate of correction is more controversial with limits falling from 10 mmol/L in 24 h and 18 mmol/L in 48 h in 2007 [43] to just 6 mmol/L in 24 h in 2013 [26]. Two-hourly checks of serum sodium are recommended to optimize rate of correction [44] with re-lowering of sodium if there is excessively rapid correction in groups at risk of osmotic demyelination [26]. In older people, special caution should be taken to avoid over-hydration due to higher incidence of cardiac failure, lower glomerular filtration rate, decreased ability to concentrate urine and higher levels of arginine vasopressin than younger people.

### 6.3. Vaptans

The development of oral vasopressin receptor antagonists (vaptans) represents a major breakthrough in the treatment of SIADH and, potentially, other forms of euvolemic or hypervolemic hyponatremia. Unlike other treatments used for SIADH (see Table 3), the vaptans target the chief pathological mechanism that accounts for the hyponatremia in this condition, an inappropriately high level of arginine vasopressin resulting in dilutional hyponatremia. Tolvaptan was first shown to be superior to placebo in two randomized controlled trials that included hyponatremic patients aged up to 100 years old with SIADH, heart failure or cirrhosis (SALT-1 and SALT-2) [45]. It was also more effective than fluid restriction at normalizing serum sodium in one small, randomized trial [46]. Serum sodium tends to fall again on discontinuation so that treatment may have to be life-long in many cases [45,47]. However, tolvaptan was not significantly better than placebo at improving mortality in the EVEREST study of patients with chronic heart failure [48] but only 10% were hyponatremic at entry. In the sub-group with hyponatremia, those given tolvaptan were more than twice as likely to experience normalization of serums sodium and reported less breathlessness [49]. More recently, tolvaptan has proven superior to placebo at raising sodium levels in SIADH in a Chinese population [50] and patients with cancer [51]. However, there remains a lack of convincing evidence of the effect on “hard” end-points such as decreased mortality or outcomes that older people value the most such as improved quality of life and better physical function [52]. A further concern is the high expense of the agents. However, one analysis of the SALT-1 and 2 suggests the reduction in length of stay in hospital is such that tolvaptan may be cost-effective [53].

### 6.4. Are Vaptans Safe in Old Age?

A major concern in treatment of older people with any new agent is the safety profile. Preclinical data give some cause for concern as these are such powerful aquaretic agents, e.g., the diuresis observed in early studies of tolvaptan averaged over 5 liters per day [54]. Nevertheless, the prevalence of serious adverse effects after treatment with tolvaptan was similar to that seen with placebo in the SALT trials [45]. Common adverse effects in the first month of therapy included thirst (14%), dry mouth (13%), weakness (9%), constipation (9%) and nausea (8%). Tolvaptan also demonstrated a good safety profile after a median 9.9 months of treatment in the EVEREST study [48], though this has to be interpreted with caution as only 10% of participants had hyponatremia. Its long-term safety profile in the setting of hyponatremia was assessed in an open-label extension of the SALT trials called SALTWATER [47]. Only 6 out of 111 patients in the study had to discontinue treatment due to probable adverse drug reactions. Subsequent studies also report a good safety profile, though follow-up was short [49,51]. However, it remains uncertain if tolvaptan would be as well tolerated in frail, elderly individuals, particularly given the potential for inappropriate prescription in dehydrated patients mis-diagnosed with SIADH. Despite the laudable inclusion of extremely elderly individuals in the SALT trials of tolvaptan (the oldest volunteer was 100 years old), mean ages of participants were just 60 years in SALT-1, 62 years in SALT-2 and 65 years in SALTWATER. The tolerability in the age group with arguably the most to gain from this treatment is therefore still unclear and warrants further evaluation in randomised controlled trials with more numerous older patients.

Overall, the vaptans currently have a role where simple and less expensive measures have failed. Yet, the potential for their use in very elderly people might be under-estimated [25], because hyponatremia is much more common in this age group, the clinical implications of any improvements in postural balance and cognition will be much greater than in a younger population, the economic burden of uncorrected hyponatremia on falls [55] and cognitive impairment [56] is huge, and the shortened life expectancy of older people with hyponatremia may make the prospect of potentially life-long treatment more acceptable to healthcare funders [25]. It remains to be seen if vaptan therapy in older patient is safe and cost-effective.

## 7. Conclusions

Hyponatremia is especially common in frail older patient groups. Even mild, chronic hyponatremia is associated with increased cognitive impairment, falls and fractures, possibly via a decrease in bone mineral density and/or bone quality. There is some evidence to show treatment of hyponatremia improves cognitive performance and postural stability. Therefore, correction of hyponatremia seems sensible in groups at high risk of falls, immobility or loss of independence from cognitive impairment. However, accurately diagnosing the causes of hyponatremia in this group is especially challenging, as hyponatremia may be multi-factorial, history hard to obtain due to cognitive or sensory impairment, and examination to determine volemic status is unreliable. Successful treatment also comes with its own challenges, both due to the potential for mis-diagnosis of underlying cause(s) and because all current therapies carry their own risks. Oral vasopressin receptor antagonists may be very helpful in this patient group, but the evidence base for their safety and efficacy on important outcomes in frail elderly groups is limited.
